# Assistierte Suizide in Bayern 2020 bis 2023 – Erste Ergebnisse aus der multikausalen Todesursachenstatistik

**DOI:** 10.1007/s00103-025-04171-w

**Published:** 2025-12-15

**Authors:** Andrea Buschner, Sabine Gleich, Benno Schäffer

**Affiliations:** 1 Bayerisches Landesamt für Statistik, Fürth, Deutschland; 2 Gesundheitsreferat LH München, München, Deutschland; 3 Institut für Rechtsmedizin der Universität München, Nußbaumstr. 26, 80336 München, Deutschland

**Keywords:** Todesbescheinigung, Gesundheitsamt, Soziodemografische Merkmale, Bevölkerungsbezogene Studie, Vorerkrankungen, Death certificate, Health department, Sociodemographic characteristics, Population-based study, Pre-existing conditions

## Abstract

**Einleitung:**

Unter bestimmten Voraussetzungen ist der assistierte Suizid (AS) in Deutschland zulässig. Bisher liegen für Deutschland Zahlen zum AS nur aus München vor. Ziel dieser Arbeit ist die Erhebung von AS-Fällen mit Wohnsitz in Bayern mit sozidemografischen Merkmalen, vorhandenen Erkrankungen und verwendeten Arzneistoffen.

**Methoden:**

Die retrospektive Analyse der Jahre 2020 bis 2023 basiert auf den Angaben der leichenschauenden Ärzte in den Todesbescheinigungen, die in die amtliche Todesursachenstatistik des Bayerischen Landesamts für Statistik eingegangen sind und eine Vollerhebung der Verstorbenen mit Wohnsitz in Bayern darstellen.

**Ergebnisse:**

In Bayern wurden insgesamt 316 AS identifiziert. Die AS-Rate stieg im Untersuchungszeitraum von 0,1 auf 1,3 Sterbefälle pro 100.000 Einwohner. 2023 machten AS fast jeden zehnten Suizid in Bayern und 0,1 % aller Sterbefälle aus. Fast zwei Drittel der AS-Fälle waren weiblich. Etwa die Hälfte war zwischen 65 und 84 Jahren alt. Fast ein Drittel litt an neurodegenerativen, etwa ein Viertel an Krebserkrankungen. Bei 7 % der Fälle wurden psychische Erkrankungen dokumentiert, davon je ein Drittel mit Demenz oder affektiven Störungen. AS bei neurodegenerativen Erkrankungen wiesen ein niedriges mittleres Sterbealter von 70,9 Jahren auf. Am häufigsten wurde mit 88,6 % Thiopental verwendet, seltener Chloroquin, Propofol oder Phenobarbital.

**Diskussion:**

Die AS-Zahlen in Bayern steigen kontinuierlich und machen mittlerweile 0,1 % aller Sterbefälle aus, was die gesamtgesellschaftliche Relevanz des Themas unterstreicht. Insbesondere für Patienten mit neurodegenerativen und Krebserkrankungen scheint AS von Bedeutung zu sein, weshalb die Suizidprävention diese vulnerablen Gruppen stärker in den Fokus nehmen sollte.

## Abstract (en)

**Introduction:**

Assisted suicide (AS) is permitted in Germany under certain conditions. To date, figures on AS in Germany are only available from Munich. The aim of this study is to collect data on AS cases involving residents of Bavaria, including sociodemographic characteristics, existing medical conditions, and drugs used.

**Methods:**

The retrospective analysis for the years 2020 to 2023 is based on the information provided in the death certificate, which leads to the official cause of death statistics of the Bavarian State Office for Statistics and represents the total of all deceased persons residing in Bavaria.

**Results:**

A total of 316 AS cases were identified in Bavaria. The AS rate increased from 0.1 to 1.3 deaths per 100,000 inhabitants during the study period. In 2023, AS accounted for almost 1 in 10 suicides in Bavaria and for 0.1% of all deaths. Almost two thirds of AS cases were female. Around half were between 65 and 84 years old. Almost a third suffered from neurodegenerative diseases and around a quarter from cancer. In addition, mental illness was documented in 7% of AS cases, with dementia and affective disorders being present in a third of these cases, respectively. AS in neurodegenerative diseases had a low mean age at death of 70.9 years. At 88.6%, thiopental was the most commonly used drug. Chloroquine, propofol, or phenobarbital were used less frequently.

**Discussion:**

The number of AS cases in Bavaria is constantly increasing and now accounts for 0.1% of all deaths, which underlines the relevance of the topic for society as a whole. As AS seems to be of particular importance for patients with neurodegenerative and cancer diseases, suicide prevention should focus on these vulnerable groups.

## Einleitung

Das Bundesverfassungsgericht stellte mit Urteil vom 26.02.2020 fest, dass ein Recht auf selbstbestimmtes Sterben besteht und dieses grundrechtlich geschützt ist. Bei freiverantwortlichen Personen schließt dieses Recht auch die Freiheit ein, sich das Leben zu nehmen [1]. Bislang fehlen konkrete Rahmenbedingungen für die Durchführung des assistierten Suizids (AS) in Deutschland. Bisher ist auch keine flächendeckende Übersicht über die Anzahl der AS verfügbar, da diese in der Todesursachenstatistik nicht getrennt von den konventionellen Suiziden (KS) ausgewiesen werden. Da der AS ein an Relevanz zunehmendes Public-Health-Thema darstellt, untersuchte eine interdisziplinäre Arbeitsgruppe aus Gesundheitsamt und Rechtsmedizin die Todesbescheinigungen (TB) und staatsanwaltschaftlichen Akten aller Münchner AS-Fälle der Jahre 2020 bis 2023. Mit der Münchner Studie konnten bundesweit erstmals die Altersverteilung und Charakteristika der Suizidenten [2], die Rolle der Sterbehilfeorganisationen und der beteiligten Ärzte [3], ein Vergleich von AS und KS [4], die Qualität vorliegender Gutachten [5] sowie für den AS eingesetzte Arzneistoffe [6] dargestellt werden.

Ziel der vorliegenden Studie ist es, für den gleichen Zeitraum Daten zu soziodemografischen Merkmalen, Erkrankungen und verwendeten Arzneistoffen bei AS-Sterbefällen für ganz Bayern vorzulegen.

## Methoden

Die vorliegende Analyse basiert ausschließlich auf den Angaben, die die leichenschauenden Ärzte im vertraulichen Teil der Todesbescheinigungen vermerkt haben und die damit in die amtliche Todesursachenstatistik eingegangen sind. Die für die Sterbeorte zuständigen Gesundheitsämter senden dem Bayerischen Landesamt für Statistik (LfStat) die Durchschläge des vertraulichen Teils der TB zu. Die Todesursachenstatistik stellt somit eine Vollerhebung der Verstorbenen mit Wohnsitz in Bayern dar.

In Bayern liegen die Todesursachen seit dem Jahr 2020 als multikausale Daten vor. Für die vorliegende Studie konnten somit erstmals für Deutschland AS-Sterbefälle hinsichtlich aller auf der TB notierten Erkrankungen untersucht werden.

Da AS in Deutschland weder meldepflichtig sind noch in der amtlichen Todesursachenstatistik mit eigenem ICD-10-Code kodiert werden, wurde vom LfStat ein anderes Verfahren zu deren Identifikation gewählt: Den oben zitierten Münchner Studien zufolge handelt es sich bei Suiziden mit bestimmten, verschreibungspflichtigen Medikamenten wie Thiopental oder Chloroquin mit hoher Sicherheit um AS [6]. Für die vorliegende Studie wurden Sterbefälle als AS definiert, wenn die leichenschauenden Ärzte eine „vorsätzliche Selbstvergiftung“ (ICD-Codes X61 und X64) mit intravenösen Anästhetika wie Thiopental (ICD-Code T41.1), Antimalariamitteln/Arzneimitteln gegen Blutprotozoen wie Chloroquin (T37.2) oder Allgemeinanästhetika wie Propofol (ICD-Code T41.2) vermerkt hatten. Seit September 2022 werden am LfStat Sterbefälle, bei denen explizit ein AS auf der TB vermerkt war, zusätzlich mit dem ICD-Code Z76.8 („Personen, die das Gesundheitswesen aus sonstigen näher bezeichneten Gründen in Anspruch nehmen“) markiert, um sie für spätere Analysen auch unabhängig von der Wahl des Arzneistoffs identifizieren zu können.

Bei den so identifizierten AS-Fällen wurden anschließend soziodemografische Merkmale wie Alter und Geschlecht, dokumentierte Erkrankungen der Suizidenten sowie die für den AS eingesetzten Arzneistoffe analysiert.

## Ergebnisse

### Gesamtzahlen assistierter und konventioneller Suizide

Im Untersuchungszeitraum 2020–2023 konnten für Bayern insgesamt 316 AS identifiziert werden (Tab. 1). Deren Zahl stieg kontinuierlich an und lag 2023 bei 177 AS-Fällen. Die KS sind von 2020 bis 2022 angestiegen und 2023 wieder leicht zurückgegangen. AS nehmen einen immer größeren Anteil an den Suiziden ein: Im Jahr 2023 war fast jeder zehnte Suizid ein AS (9,8 %), bezogen auf alle Sterbefälle lag der Anteil der AS in diesem Jahr bei etwa einem Promille (0,1 %).

**Tab. 1:** Sterbefälle und (assistierte) Suizide in Bayern im Zeitvergleich (2020–2023)

Jahr	Anzahl der Sterbefälle insgesamt	Anzahl aller Suizide (AS + KS)	Anzahl der AS	Anteil der AS an allen Suiziden (in %)	Anteil der AS an allen Sterbefällen (in %)
2020	143.367	1523	9	0,6	0,006
2021	147.984	1600	38	2,4	0,026
2022	152.417	1811	92	5,1	0,060
2023	146.475	1799	177	9,8	0,121
*Insgesamt*	*590.243*	*6733*	*316*	*4,7*	*0,054*

*AS* assistierte Suizide, *KS* konventionelle Suizide

Der leichte Anstieg aller Suizidsterbefälle sowie die deutliche Zunahme der AS lässt sich auch bei den rohen Suizidraten erkennen (Tab. 2). Für die AS konnte im Zeitverlauf von 2020 bis 2023 ein durchgehender Anstieg der Suizidraten festgestellt werden (von 0,1 auf 1,3 Sterbefälle pro 100.000 Einwohner).

**Tab. 2:** Suizidraten pro 100.000 Einwohner in Bayern im Zeitvergleich (2020–2023)

Jahr	Alle Suizide		KS		AS	
	Rohe Rate	Veränderung zum Vorjahr	Rohe Rate	Veränderung zum Vorjahr	Rohe Rate	Veränderung zum Vorjahr
2020	11,6	–	11,5	–	0,1	–
2021	12,2	+5,2 %	11,9	+3,5 %	0,3	+200,0 %
2022	13,6	+11,5 %	13,0	+9,2 %	0,7	+133,3 %
2023	13,4	−1,5 %	12,1	−6,9 %	1,3	+85,7 %

*AS* assistierte Suizide, *KS* konventionelle Suizide

### Soziodemografische Merkmale der AS-Fälle

**Abb. 1:**
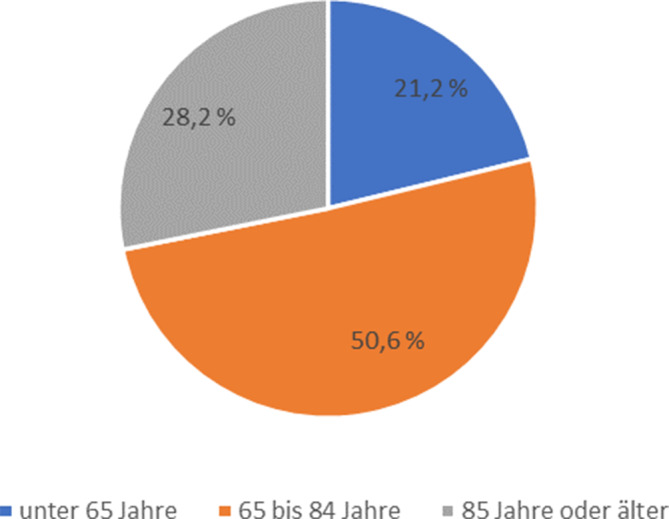
Altersverteilung der durch assistierten Suizid Verstorbenen in Bayern (2020–2023). *n* = 316 AS

AS-Fälle waren zu fast 2 Dritteln weiblich (202 Fälle, 63,9 %). Deren mittleres Alter betrug 76,3 Jahre (Median: 79,0, Spannweite: 23–100), das mittlere Alter der 114 männlichen AS-Fälle 72,9 Jahre (Median: 77,5, Spannweite: 20–96). Etwa die Hälfte der AS-Fälle war zwischen 65 und 84 Jahren alt (Abb. 1).

**Abb. 2:**
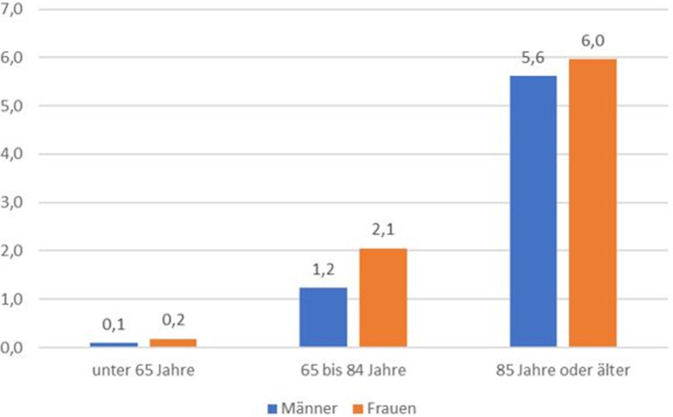
Durchschnittliche, jährliche altersspezifische Sterberaten für assistierte Suizide in Bayern pro 100.000 Einwohner der jeweiligen Alters‑/Geschlechtsgruppe (2020–2023). *n* = 316 AS

Die Betrachtung der altersspezifischen Raten für den gesamten Zeitraum 2020 bis 2023 ergab, dass die Geschlechtsunterschiede der AS-Fälle nicht ganz so deutlich ausgeprägt waren, wie sie bei dem Ergebnis der absoluten Geschlechtsverteilung von fast 2/3 Frauen zu 1/3 Männern zu erwarten gewesen wären (Abb. 2). Werden alle AS-Fälle im Alter von 85 Jahren oder älter betrachtet, so lagen pro 100.000 Männer dieser Altersgruppe 5,6 AS-Fälle vor, während sich der Wert bei Frauen dieses Alters auf 6,0 belief. In der mittleren Altersgruppe von 65 bis 84 Jahren waren die Geschlechtsunterschiede etwas stärker ausgeprägt.

**Abb. 3:**
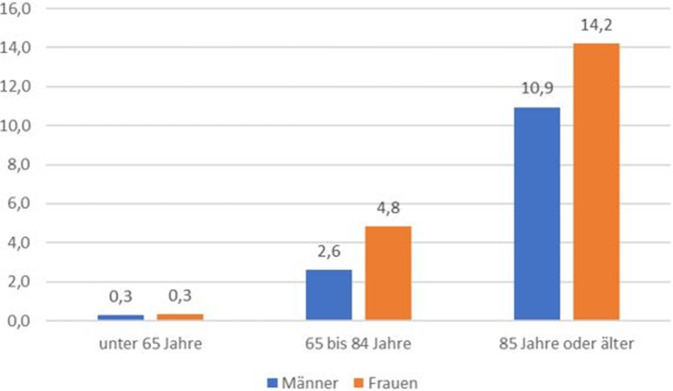
Altersspezifische Sterberate für assistierte Suizide in Bayern pro 100.000 Einwohner der jeweiligen Alters‑/Geschlechtsgruppe (ausschließlich 2023). *n* = 177 AS

Die über den gesamten Untersuchungszeitraum nicht ganz so deutlich ausgeprägten Geschlechtsunterschiede sind vor allem der Tatsache geschuldet, dass 2020 und 2021 etwa gleich viele Männer wie Frauen einen AS verübt haben, während sich in den beiden Folgejahren 2022 und 2023 eine deutliche Verteilung zugunsten der Frauen einstellte:

Im Jahr 2023 wählten 14,2 Frauen pro 100.000 Einwohnerinnen im Alter von 85 Jahren oder älter einen AS (Abb. 3), der Wert für die Männer dieser Altersgruppe lag bei 10,9. Die altersspezifische Rate für AS war in dieser Altersgruppe somit bei Frauen um das 1,3-Fache höher als bei Männern.

### Erkrankungen der AS-Fälle

Die bestehenden Erkrankungen der AS-Fälle können Tab. 3 entnommen werden: Fast ein Drittel (30,7 %) litt an einer neurodegenerativen Erkrankung, wie beispielsweise amyotrophe Lateralsklerose (ALS), Morbus (M.) Parkinson, multiple Sklerose (MS), Lähmungssyndrome sowie Krankheiten des peripheren Nervensystems. Bösartige Neubildungen waren bei etwa einem Viertel (26,6 %) der AS-Fälle dokumentiert, am häufigsten solche der Verdauungsorgane, gefolgt von Brust- und Prostatakrebs. Psychische und Verhaltensstörungen wurden bei 7,3 % der AS-Fälle dokumentiert, wobei in etwa einem Drittel dieser Fälle eine Demenz (*n* = 8) und ebenso häufig eine affektive Störung vorlag (*n* = 8).

**Tab. 3:** Ausgewählte Erkrankungen bei durch assistierte Suizide (AS) Verstorbenen in Bayern (2020–2023)

Erkrankungen	ICD-10-Code-Bereich	Anzahl der AS mit Komorbidität aus dem Bereich …	Anteil der AS mit Komorbidität aus dem Bereich … an allen AS (in %)	Anteil der AS mit Komorbidität aus dem Bereich … an der jeweiligen Obergruppe (in %)
*Bösartige Neubildungen (BN)*	*C00-C07*	*84*	*26,6*	–
BN der Verdauungsorgane	C15-C26	31	9,8	36,9
BN der Lunge	C34	7	2,2	8,3
BN der Brust	C50	15	4,8	17,9
BN der Prostata	C61	12	3,8	14,3
*Endokrine, Ernährungs- und Stoffwechselkrankheiten*	*E00-E90*	*14*	*4,4*	–
*Psychische und Verhaltensstörungen*	*F00-F99*	*23*	*7,3*	–
Demenz	F00-F03	8	2,5	34,8
Affektive Störungen	F30-F39	8	2,5	34,8
*Neurodegenerative Erkrankungen*	*G00-G99*	*97*	*30,7*	–
Amyotrophe Lateralsklerose (ALS)	G12.2	18	5,7	18,6
Morbus Parkinson	G20-G21	17	5,4	17,5
Multiple Sklerose (MS)	G35	9	2,9	9,3
Episodische und paroxysmale Krankheiten des Nervensystems	G40-G47	9	2,9	9,3
Epilepsie	G40-G41	4	1,3	4,1
Polyneuropathien und sonstige Krankheiten des peripheren Nervensystems	G60-G64	14	4,4	14,4
Zerebrale Lähmung und sonstige Lähmungssyndrome	G80-G83	15	4,8	15,5
*Krankheiten des Kreislaufsystems*	*I00-I99*	*114*	*36,1*	–
Chronische ischämische Herzkrankheit	I25	16	5,1	14,0
*Krankheiten des Atmungssystems*	*J00-J99*	*28*	*8,9*	–
Chronische Krankheiten der unteren Atemwege (u. a. COPD und Asthma)	J40-J47	21	6,7	75,0
*Krankheiten des Verdauungssystems*	*K00-K93*	*18*	*5,7*	–
*Erkrankungen des Muskel-Skelett-Systems und des Bindegewebes*	*M00-M99*	*59*	*18,7*	–
Arthrose und Spondylose	M15-M19, M47	19	6,0	32,2
Systemkrankheiten des Bindegewebes	M30-M36	10	3,2	16,9
Krankheiten der Wirbelsäule und des Rückens	M40-M54	18	5,7	30,5
Krankheiten der Weichteilgewebe	M60-M79	5	1,6	8,5
Osteoporose	M80-M81	15	4,8	25,4
*Krankheiten des Urogenitalsystems*	*N00-N99*	*17*	*5,4*	*28,8*

*COPD* chronisch obstruktive Lungenerkrankung

Mehrfachnennungen möglich; Datenbasis: *n* = 316 AS

Erkrankungen, die ebenfalls häufig auf den TB der AS-Fälle vermerkt waren, waren Krankheiten des Kreislaufsystems (36,1 %), Erkrankungen des Muskel-Skelett-Systems und des Bindegewebes (18,7 %) sowie Krankheiten des Atmungssystems (8,9 %), wie beispielsweise die chronisch obstruktive Lungenerkrankung (COPD). Insgesamt 32 TB enthielten über den Suizid und die verwendeten Arzneistoffe hinaus keine weiteren Erkrankungen.

### Sterbealter der AS-Fälle in Abhängigkeit von vorliegenden Erkrankungen

Ein vergleichsweise niedriges mittleres Sterbealter mit 70,9 Jahren (Median: 72; Spannweite: 20–95) zeigten AS-Fälle mit neurodegenerativen Erkrankungen. AS-Fälle ohne neurodegenerative Erkrankungen wiesen ein mittleres Sterbealter von 77,0 Jahren auf (Median: 80; Spannweite: 22–100). Im Detail war das Durchschnittsalter bei Durchführung des AS in Jahren wie folgt: ALS 65,9 (Median: 64,5; Spannweite: 53–83), MS 65,9 (Median: 68; Spannweite: 51–79) und M. Parkinson 74,9 (Median: 78; Spannweite: 58–88).

Im Mittel waren bei den AS-Sterbefällen 4,3 Erkrankungen auf der TB vermerkt, bei Frauen mit 4,5 etwas mehr als bei Männern (4,0; Tab. 4). Mit zunehmendem Alter erhöhte sich die mittlere Zahl an Erkrankungen.

**Tab. 4:** Durchschnittliche Anzahl der Erkrankungen auf der Todesbescheinigung (TB) bei Sterbefällen durch assistierten Suizid in Bayern

Anzahl der Erkrankungen auf der TB	Gesamt	Männer	Frauen
**Insgesamt**	**4,3**	**4,0**	**4,5**
*Altersgruppen*			
Unter 65 Jahren	3,2	3,0	3,4
65 bis 84 Jahre	4,3	4,5	4,3
85 Jahre oder älter	5,1	4,3	5,5

### Eingesetzte Arzneistoffe

Bei der Durchführung der AS kamen verschiedene Arzneistoffe zum Einsatz. Am häufigsten wurden für diesen Zweck intravenöse Anästhetika wie Thiopental (280 Fälle, 88,6 %) oder Allgemeinanästhetika wie Propofol (8 Fälle, 2,5 %) eingesetzt. Eine Kombination aus Antimalariamitteln/Arzneimitteln gegen Blutprotozoen und Benzodiazepinen (Chloroquin, Diazepam) fand bei 15 Fällen (4,7 %) Anwendung. Darüber hinaus wurden bei 13 AS-Fällen andere Arzneistoffe wie Barbiturate (Phenobarbital) oder Neuroleptika eingesetzt.

## Diskussion

### Gesamtzahlen assistierter und konventioneller Suizide

Die Anzahl der Suizide in Deutschland hat sich seit den 1980er-Jahren nahezu halbiert und hält sich in den letzten 10 Jahren bundesweit relativ konstant auf einem Niveau um die 9000 bis 10.000 Fälle pro Jahr [7]. Auch in Bayern sind die Suizidzahlen seit 1980 gesunken, bis zum Jahr 2017 um etwa 29 %. Dabei ist der Rückgang bei Frauen mit über 50 % deutlich ausgeprägter als bei Männern mit einem Rückgang von etwa 18 %. Der Rückgang der Suizide seit 1980 wird auf mehrere Faktoren zurückgeführt, u. a. verbesserte Diagnostik und Behandlung von Depressionen [8]. Für die Stagnation in den letzten Jahren werden soziale Isolation, wirtschaftliche Krisen und gesellschaftliche Veränderungen als mögliche Ursachen diskutiert [8].

Die vorliegende Analyse der bayerischen Daten aus den Jahren 2020 bis 2023 zeigte ebenfalls relativ konstante Raten für KS. Dem steht jedoch eine starke Zunahme des AS in Bayern seit 2020 gegenüber. Dieser Trend wird auch in der Schweiz beobachtet: 2023 wählten dort im Vergleich zum Vorjahr +6,8 % mehr Männer und +9,6 % mehr Frauen einen AS, um aus dem Leben zu scheiden [9, 10]. Die Zunahme der AS-Fälle in Bayern könnte mit einer wachsenden Bekanntheit in der Bevölkerung zusammenhängen, die unter anderem durch medienwirksame Strafverfahren gegen Ärzte, die Suizidassistenz geleistet haben, gefördert wird.^1^

In Deutschland betrug im Jahr 2023 die Suizidrate 12,2 Suizide pro 100.000 Einwohner [11]. Im europäischen Vergleich lag das Bundesgebiet in den letzten Jahren im unteren Mittelfeld [12]. Die bayerischen Suizidrate lag in der vorliegenden Studie 2023 mit 13,4 pro 100.000 Einwohnern etwas darüber. Die Ursachen dafür sind nicht abschließend geklärt, jedoch ist bekannt, dass die Suizidrate in Bayern seit Jahren über dem Bundesdurchschnitt liegt [8]. Der Anteil der AS lag im Jahr 2023 bei etwa einem Promille (0,1 %) aller Sterbefälle in Bayern und befindet sich damit im internationalen Vergleich (Schweiz, Belgien, Niederlande, Luxemburg, Kanada, Kolumbien, Neuseeland und Teile der USA und Australiens) auf einem niedrigen Niveau (0,1–5,1 % der Sterbefälle; [13, 14]). Die Zahlen aus dem Ausland können jedoch nicht direkt mit den hiesigen Zahlen verglichen werden, da sie neben AS auch die in Deutschland verbotene aktive Sterbehilfe (Euthanasie) umfassen können [2].

### Soziodemografische Merkmale der AS-Fälle

Auf Bundesebene zeigen sich in Deutschland bei KS erhebliche Unterschiede in der Verteilung nach Geschlecht und Alter. So begehen deutsche Männer 2023 mit einer Rate von 17,9 pro 100.000 Einwohner nahezu 3‑mal so häufig Suizid wie Frauen, deren Rate bei 6,6 pro 100.000 Einwohnerinnen liegt [7]. AS hingegen sind in Bayern ein weibliches Phänomen: auch bei Berücksichtigung der Altersstruktur der Gesellschaft verüben mehr Frauen als Männer einen AS. Dies steht in Einklang mit dem Geschlechterverhältnis bei AS in der Münchner Studie [4], den Zahlen aus der Schweiz [9, 10] und aus Österreich [15]. Ein möglicher Erklärungsansatz für den Überhang von Frauen beim AS könnte damit zusammenhängen, dass die für den AS verwendete Suizidmethode der Vergiftung, auch beim KS tendenziell häufiger von Frauen gewählt wird [16]. Dem steht entgegen, dass international eher Männer Sterbehilfe (aktive und/oder AS) in Anspruch zu nehmen scheinen [14]. Ein möglicher weiterer Grund für den Überhang von Frauen beim AS könnte in der Lebenssituation der Verstorbenen liegen: Frauen kommen eher in Lebensumstände, in denen ein AS in Erwägung gezogen wird. Sie haben eine höhere Lebenserwartung [17] und werden damit häufiger chronisch krank und multimorbid. Zudem sind sie in dieser Situation dann auch häufig noch allein (verwitwet; [18]).

Wie in München hat sich auch in ganz Bayern beim AS das deutlich ausgeprägte Geschlechterverhältnis von Männern zu Frauen von 1:2 erst in den Jahren 2022 und 2023 eingestellt. Während in München das Geschlechterverhältnis von Männern zu Frauen im Jahr 2020 und 2021 noch ausgeglichen war, dominierten Frauen beim AS ab 2022 (7 Männer versus 12 Frauen) und in 2023 (13 Männer versus 27 Frauen). In der französischsprachigen Schweiz zeigte sich während der akuten Phase der COVID-19-Pandemie in den Jahren 2020 und 2021 eine Abnahme der AS-Rate bei Frauen [19]. In der vorliegenden Studie zeigten sich in der Hochphase der Pandemie 2020 und 2021 bei Frauen niedrige AS-Zahlen, wobei dieser Zeitraum mit der geänderten Rechtslage des AS in Deutschland zusammenfällt.

Der Überhang an Frauen beim AS in Bayern ist am stärksten in der mittleren Altersgruppe (65 bis 84 Jahre) ausgeprägt. Die mittlere Altersgruppe weist sowohl in Bayern als auch international die höchste Anzahl an AS-Fällen auf [14]. Dies könnte darauf hindeuten, dass Personen unter 65 Jahren tendenziell weniger den Wunsch nach AS haben, möglicherweise aufgrund noch bestehender Selbstständigkeit und sozialer Integration oder schlichtweg deshalb, weil unter 65 Jahren die als typisch für den AS angesehenen Erkrankungen (ALS, M. Parkinson, MS und Krebserkrankungen) noch nicht vorlagen. Im Gegensatz dazu könnte AS bei Personen ab 85 Jahren häufig nicht mehr durchführbar sein, da z. B. schwere Demenz als Ausschlussgrund für eine eigenverantwortliche Entscheidung gelten kann.

### Erkrankungen der AS-Fälle

In Bayern beim AS häufig vorkommende Vorerkrankungen waren neurodegenerative Erkrankungen, bösartige Neubildungen und Erkrankungen des Muskel-Skelett-Systems. In anderen Ländern gehören Krebserkrankungen und ALS ebenfalls zu den häufigsten Vorerkrankungen bei Personen, die medizinische Unterstützung beim Sterben in Anspruch nehmen [13]. In der vorliegenden Studie waren kardiovaskuläre Erkrankungen ebenfalls sehr häufig, jedoch in einem Drittel der Fälle als Folge des Suizids (z. B. als Herzstillstand) notiert. Zudem treten Herz-Kreislauf-Erkrankungen gerade mit höherem Alter bei einer Vielzahl an Verstorbenen auf [20] und müssen daher nicht notwendigerweise ein relevantes Motiv für die Wahrnehmung von AS sein.

Bei 7 % der AS-Fälle wurden psychische Erkrankungen (insbesondere Demenz und affektive Störungen) dokumentiert, mithin potenziell die Freiverantwortlichkeit beeinträchtigende Erkrankungen. Das Bundesverfassungsgericht hat in seinem Urteil vom 26.02.2020 Voraussetzungen für die Freiverantwortlichkeit der Suizidentscheidung formuliert. Insbesondere muss der Sterbewillige in der Lage sein, seinen Willen frei und unbeeinflusst von einer akuten psychischen Störung zu bilden und entsprechend dieser Einsicht zu handeln. Hierbei können die Kriterien zur Einwilligungsfähigkeit als Orientierung dienen. Die Beurteilung sollte höchsten Sorgfaltsmaßstäben genügen [1]. Im Münchner Kollektiv hatte sich gezeigt, dass gerade bei dieser vulnerablen Klientel in weniger als der Hälfte der Fälle Fachgutachter aus Psychiatrie oder Psychologie beauftragt worden waren [5]. Inwieweit die vergleichsweise niedrige Zahl an AS-Fällen mit psychischen Erkrankungen in der vorliegenden Studie auf Qualitätsprobleme bei der Leichenschau, dem Ausfüllen von TB bzw. der finalen Erstellung der Todesursachenstatistik zurückzuführen ist oder möglicherweise den tatsächlichen Ausschluss akut psychisch erkrankter und dadurch nicht freiverantwortlich handelnder Personen widerspiegelt, kann mit den vorliegenden Daten nicht geprüft werden.

### Sterbealter der AS-Fälle in Abhängigkeit von vorliegenden Erkrankungen

Die Analyse der bayerischen AS-Fälle zeigt, dass neurodegenerative Erkrankungen wie ALS und MS mit einem vergleichsweise niedrigen mittleren Sterbealter von jeweils 65,9 Jahren verbunden sind. Laut Literatur liegt das durchschnittliche Erkrankungsalter bei ALS zwischen 58 und 60 Jahren, wobei die durchschnittliche Überlebenszeit von der Diagnose bis zum Tod lediglich 3 bis 4 Jahre beträgt [21]. Patienten mit ALS nehmen AS auch häufiger in Anspruch als Krebspatienten [22]. Zudem könnte im Einzelfall auch ein besonderer Wunsch zur Wahrnehmung von AS in einem frühen Krankheitsstadium bestehen. Hier spielen unter anderem die Krankheitslast selbst oder depressive Symptome eine Rolle [23]. In der Literatur wird das mittlere Sterbealter für MS generell mit 64,8 Jahren angegeben [24], was in etwa den vorliegenden bayerischen Daten mit einem durchschnittlichen Alter von 65,9 Jahren bei Durchführung des AS entspricht (s. oben). Da AS insbesondere für Patienten mit ALS und MS von Bedeutung zu sein scheint [22, 25] und laut bayerischen Daten in einem vergleichsweise jungen Lebensalter verübt wird, sollte die Suizidprävention dieser vulnerablen Gruppen besonders in den Fokus rücken.

### Eingesetzte Arzneistoffe

International wird laut Literatur eine Vielzahl von intravenös oder oral verabreichten Medikamenten zur Sterbehilfe eingesetzt. Intravenös kommen insbesondere Barbiturate wie Thiopental und Allgemeinanästhetika wie Propofol zum Einsatz, Letzteres oft in Kombination mit einem Muskelrelaxans wie Rocuronium. Oral werden am häufigsten Barbiturate wie Pentobarbital, allein oder in Kombination mit einem Opioid wie Morphin, sowie einem Prokinetikum wie Metoclopramid verwendet. Chloroquin scheint international nicht eingesetzt zu werden [26, 27].

Für Deutschland lagen bisher kaum Daten zu den eingesetzten Arzneistoffen vor [28]. Die Durchführung der AS in Bayern zeigt eine klare Präferenz für das intravenös applizierte Thiopental, das in 280 AS-Fällen (88,6 %) zum Einsatz kam. Dies hatte sich bereits in München gezeigt [6]. Die Verwendung von Phenobarbital und Propofol in einzelnen bayerischen AS-Fällen verdeutlicht die Vielfalt der Arzneistoffe, die beim AS in Betracht gezogen werden. Chloroquin wurde selten eingesetzt und sollte nach Ansicht der Autoren der Münchner Studie aufgrund seiner häufigen Komplikationen für den AS auch nicht mehr verwendet werden. 13 der 15 bayerischen Chloroquin-Fälle wurden in München registriert [6].

## Limitationen

Für die vorliegende Studie wurden die Daten der Todesursachenstatistik ausgewertet, die ausschließlich auf den Informationen in den TB beruhen. Die Datenqualität hing somit von der Qualität der TB ab [29]. Die der Münchner Studien weichen davon leicht ab, da die dort gesichteten Akten und Unterlagen der Staatsanwaltschaft deutlich umfassendere Daten enthielten (z. B. Ergebnis des polizeilichen Todesermittlungsverfahrens). Zudem liegt in der Todesursachenstatistik kein separater ICD-Code für einen AS vor. Während im Rahmen der vorliegenden Studie AS bis September 2022 lediglich auf Basis der eingesetzten Arzneimittel bei einem Suizid identifiziert werden können, wurden AS ab diesem Zeitpunkt mit einem eindeutigen zusätzlichen ICD-Code versehen, wenn der AS vom leichenschauenden Arzt konkret benannt wurde. Über diese Markierung der AS-Fälle konnten im gesamten Untersuchungszeitraum 15 zusätzliche Fälle identifiziert werden, die auf Basis der verabreichten Medikamente nicht als AS definiert worden wären. Durch die Auswahl der Untersuchungsgruppe über die eingesetzten Arzneistoffe kann jedoch nicht ausgeschlossen werden, dass es sich nicht auch um KS unter Verwendung von Chloroquin gehandelt hat. Insgesamt gab es im Untersuchungszeitraum nur 15 Chloroquin-Fälle, weshalb keine größere Verzerrung durch potenzielle KS-Fälle aufgrund von Chloroquin zu erwarten ist. Bei der Allgemeinbevölkerung ist beim Einsatz von Thiopental, Propofol oder Phenobarbital nicht von einem KS auszugehen, da diese Arzneistoffe intravenös appliziert werden.

## Fazit

Für die vorliegende Studie konnten erstmals für Deutschland AS-Sterbefälle hinsichtlich aller auf der TB notierten Erkrankungen untersucht werden. Dies verdeutlicht das enorme Potenzial der multikausalen Todesursachenstatistik für populationsbezogene Studien. In 12 weiteren Bundesländern liegen die multikausalen Daten für verschiedene Zeiträume in den statistischen Landesämtern vor, wurden aber bislang nicht im Hinblick auf AS ausgewertet. Ebenso zeigt die vorliegende Untersuchung erstmals für ein deutsches Bundesland, dass die AS-Zahlen in Bayern konstant steigen und mittlerweile 0,1 % aller Sterbefälle ausmachen, was die gesamtgesellschaftliche Relevanz des Themas betont. Da AS insbesondere für Patienten mit neurodegenerativen und Krebserkrankungen von Bedeutung zu sein scheinen, sollte die Suizidprävention diese vulnerablen Gruppen besonders in den Fokus nehmen. Das von der letzten Bundesregierung initiierte Suizidpräventionsgesetz (vgl. Bundestagsdrucksache 20/14987) hätte auch eine Beobachtung der AS-Zahlen etabliert, wurde jedoch nach Ablauf der Wahlperiode nicht weiter forciert. Die Etablierung einer bundesweit einheitlichen Datengrundlage wäre aber wichtig für Prävention und Versorgung. In der Todesursachenstatistik sollten AS und KS künftig bundesweit getrennt und mit jeweils einheitlichem ICD-Code ausgewiesen werden. Entwicklungen der Suizidzahlen könnten damit differenziert beobachtet werden. Maßnahmen der Suizidprävention könnten zudem passgenauer gestaltet werden, da für KS und AS gefährdete Personen unterschiedliche Präventions- und Interventionsbedarfe aufweisen.

## Data Availability

Die während der vorliegenden Studie analysierten Datensätze sind aufgrund von Geheimhaltungsrichtlinien im statistischen Verbund derzeit noch nicht öffentlich zugänglich.

## Footnotes

1 Siehe hierzu das viel beachtete Gerichtsverfahren vor dem Landgericht (LG) Essen (Urteil vom 01.02.2024 – 32 Ks-70 Js 354/20-5/23) und dem LG Berlin I (Urteil vom 08.04.2024 – [540 Ks] 278 Js 405/21 [2/23]).
